# Identification of Host-Immune Response Protein Candidates in the Sera of Human Oral Squamous Cell Carcinoma Patients

**DOI:** 10.1371/journal.pone.0109012

**Published:** 2014-10-01

**Authors:** Yeng Chen, Siti Nuraishah Azman, Jesinda P. Kerishnan, Rosnah Binti Zain, Yu Nieng Chen, Yin-Ling Wong, Subash C. B. Gopinath

**Affiliations:** 1 Department of Oral Biology & Biomedical Sciences, Faculty of Dentistry, University of Malaya, Kuala Lumpur, Malaysia; 2 Oral Cancer Research and Coordinating Center, Faculty of Dentistry, University of Malaya, Kuala Lumpur, Malaysia; 3 Institute for Research in Molecular Medicine, Universiti Sains Malaysia, Georgetown, Penang, Malaysia; 4 Department of Oro-Maxillofacial and Medical Science, Faculty of Dentistry, University of Malaya, Kuala Lumpur, Malaysia; 5 Chen Dental Specialist Clinic, Kueh Hock Kui Commercial Centre, Jalan Tun Ahmad Zaidi Adruce, Kuching, Sarawak, Malaysia; Duke Cancer Institute, United States of America

## Abstract

One of the most common cancers worldwide is oral squamous cell carcinoma (OSCC), which is associated with a significant death rate and has been linked to several risk factors. Notably, failure to detect these neoplasms at an early stage represents a fundamental barrier to improving the survival and quality of life of OSCC patients. In the present study, serum samples from OSCC patients (n = 25) and healthy controls (n = 25) were subjected to two-dimensional gel electrophoresis (2-DE) and silver staining in order to identify biomarkers that might allow early diagnosis. In this regard, 2-DE spots corresponding to various up- and down-regulated proteins were sequenced via high-resolution MALDI-TOF mass spectrometry and analyzed using the MASCOT database. We identified the following differentially expressed host-specific proteins within sera from OSCC patients: leucine-rich α2-glycoprotein (LRG), alpha-1-B-glycoprotein (ABG), clusterin (CLU), PRO2044, haptoglobin (HAP), complement C3c (C3), proapolipoprotein A1 (proapo-A1), and retinol-binding protein 4 precursor (RBP4). Moreover, five non-host factors were detected, including bacterial antigens from *Acinetobacter lwoffii, Burkholderia multivorans, Myxococcus xanthus, Laribacter hongkongensis*, and *Streptococcus salivarius*. Subsequently, we analyzed the immunogenicity of these proteins using pooled sera from OSCC patients. In this regard, five of these candidate biomarkers were found to be immunoreactive: CLU, HAP, C3, proapo-A1 and RBP4. Taken together, our immunoproteomics approach has identified various serum biomarkers that could facilitate the development of early diagnostic tools for OSCC.

## Introduction

Oral cancer represents the sixth most prevalent cancer in the world. Among the different types of oral cancer, oral squamous cell carcinoma (OSCC) arising from the oral mucosa accounts for more than 90% of these malignancies. Thus, OSCC is the most common malignancy affecting the head and neck region. Notably, there are nearly 300,000 new cases of oral cancer reported annually [1], [2], and it was estimated that approximately 128,000 oral cancer patients died worldwide in 2008 [3]. Therefore, despite recent advances in the diagnosis and treatment of oral cancer (e.g., chemotherapy, radiotherapy, and surgical therapy) the survival rate of OSCC patients has remained less than 60% [4], [5]. A fundamental barrier in improving the survival of OSCC patients is the fact that these malignancies often remain undetected until the later stages. In this regard, it was reported that a several-month delay in diagnosis could reduce the chance of survival from 80% to 40% [6]. Thus, in order to prevent the high OSCC-related mortality rate, recent attention has been focused on identifying potential diagnostic molecular markers (e.g., cell cycle regulators) that might represent biological predictors of oral cancer [7]. Moreover, OSCC has been linked to several risk factors, including various bacterial pathogens [8], which might be useful for detecting OSCC.

Recent studies have indicated that early diagnosis, lifestyle modification, and effective treatment can prevent more than two-thirds of OSCC-related mortalities. However, currently available diagnostic methods do not allow for the detection of oral cancer in the early stages. To visualize malignant lesions in the oral cavity, different microscopic methods are available, which make use of various techniques, including autofluorescence, chemiluminescence, or dye-based tissue staining. However, due to the low sensitivity and specificity of these diagnostic strategies, clinicians generally use biopsies to detect OSCC [9]. Nevertheless, successful diagnosis through tissue biopsy is highly dependent on acquiring whole and complete tissue samples from patients for examination. In this regard, biopsies harvested from oral cancer patients are often associated with the soft tissues that surround the cancer tissue. In addition, oral cancer frequently involves the development of multiple primary tumors. Indeed, the occurrence of a second primary tumor is 3–7% higher per year in oral cancer when compared to other malignancies [10]. Therefore, the identification of suitable and reliable OSCC biomarkers is essential for achieving early detection and treatment, which can reduce mortality rates in OSCC patients. In this respect, antibody-based diagnostic tests that recognize specific tumor-associated antigens in cancer sera might represent a valid methodology [11].

Proteomic analysis allows the identification and quantification of proteins and peptides in biological samples [12]. However, through this approach, numerous post-translational forms of protein regulation, including regulating enzymes and low abundance proteins may remain undetected. Thus, in the present investigation, we employed an immunoproteomic approach and pooled human antibodies to detect host-specific response proteins in OSCC patients. Specifically, we used a well-characterized analytical platform combining two-dimensional gel electrophoresis (2-DE) and mass spectrometry (MS) to identify biomarkers in unfractionated sera from OSCC patients and normal controls. This immunoproteomics approach can be used to identify antigens targeted by the immune system in sera during disease progression. In addition, the immune responses are known to be involved in the mechanism of carcinogenesis [13]. Therefore, our comparative analyses revealed distinct OSCC biomarkers that might promote the development of specific diagnostic tests for early detection of oral cancer.

## Materials and Methods

### Serum samples

Twenty-five serum samples from OSCC patients were obtained from the Oral Cancer Research and Coordinating Center (OCRCC) at the University of Malaya (Kuala Lumpur). Additionally, 25 control serum samples were acquired from healthy individuals. All samples were collected with the verbal consent of patients, and the Dental Faculty at the University of Malaya and the Universiti Sains Malaysia Medical Ethics Committee (Ref: USMKK/PPP/JEPeM [213.3(09)]) approve this consent procedure. We obtained the permission from the above committee and they have cleared all the approval and having the record. This study was also conducted in accordance with International Conference on Harmonisation–Good Clinical Practice (ICH–GCP) guidelines and the Declaration of Helsinki.

### Two-dimensional gel electrophoresis (2-DE)

We performed 2-DE as previously described [14]. Briefly, unfractionated human serum samples (10 μl) were lysed, rehydrated in lysis buffer (2M thiourea, 8M urea, 4% CHAPS, 1% dithreitol, and 2% pharmalyte), and subjected to isoelectric focusing in 13-cm rehydrated precast immobilized dry strips (pH 4–7; GE Healthcare, Sweden). Sodium dodecyl sulfate–polyacrylamide gel electrophoresis (SDS–PAGE) was performed using 8–18% gradient polyacrylamide gels in the presence of SDS for the second dimension separation. Gel silver staining was performed as previously described [15]. Silver staining and Coomassie Brilliant Blue staining for MS were conducted using slightly modified published methods [16].

### Differential image acquisition and statistical analysis

The ImageScanner III (GE Healthcare, Sweden) was used to capture and store 2-DE gel images. PD-Quest 2-D gel analysis software (version 8.0.1, Bio-Rad) was employed to evaluate the differentially expressed protein spots. Identical spots were matched in the serial gels and normalized by correcting for spot quantification values and gel-to-gel variation unrelated to expression changes. For the normalization method, we used total densities from the gel images (i.e., raw quantity of each gel spot was divided by the total quantity of all spots within the gel). All protein concentration values are presented as means of percentage volume (% volume) ± standard deviations (SD). The student’s t-test and one-way analysis of variance (ANOVA) were used to analyze differences between patients and controls. *P*-values less than 0.05 (p<0.05) were considered as statistically significant.

### Mass spectrometry analysis and database search

Spots of interest were excised and subjected to in-gel tryptic digestion using a commercially available kit (Calbiochem, Germany). MS analysis and database searches were performed at the Proteomic Center within the Faculty of Biological Sciences at the National University of Singapore. Digested peptides were mixed with 1.2 μl of CHCA matrix solution (5 mg/ml of cyano-4-hydroxy-cinnamic acid in 0.1% trifluoroacetic acid [TFA] and 50% acetonitrile [ACN]) and spotted onto MALDI target plates. An ABI 4800 Proteomics Analyzer MALDI-TOF/TOF Mass Spectrometer was used for spectra analysis (Applied Biosystems, USA), and the MASCOT search engine (version 2.1; Matrix Science, UK) was employed for database searches. In addition, GPS Explorer software (version 3.6; Applied Biosystems, USA) was utilized along with MASCOT to identify peptides and proteins. Search parameters allowed for N-terminal acetylation, C-terminal carbamidomethylation of cysteine (fixed modification), and methionine oxidation (variable modification). Peptide and fragment mass tolerance were set to 100 ppm and ±0.2 Da, respectively. The peptide mass fingerprinting (PMF) parameters were set as follows: one missed cleavage allowed in trypsin digest; monoisotropic mass value; ±0.1 Da peptide mass tolerance; and 1+ peptide charge state.

Peptides were initially identified using the ProteinPilot proteomics software on the Mass Spectrometer (Applied Biosystems, USA). A score reflecting the relationship between theoretically and experimentally determined masses was calculated and assigned. Analyses were conducted using the International Protein Index (http://www.ebi.ac.uk/IPI) and NCBI Unigene human databases (version 3.38). A total of 100,907 entries were searched, and a score of >82 was considered as significant in the MASCOT NCBI database.

### Immunoblotting

Our 2-DE gel immunoblotting protocol was organized into four categories: (1) normal sera probed with normal sera, (2) normal sera probed with OSCC sera, (3) OSCC sera probed with normal sera, and (4) OSCC sera probed with OSCC sera. After running the 2-DE gels, they were transferred onto nitrocellulose membranes using the Multiphor II Novablot semi-dry system (GE Healthcare, Sweden). Membranes were blocked with SuperBlock (Pierce, USA) and washed three times with Tris-buffered saline (TBS)–Tween-20. The membranes were subsequently incubated overnight (4°C) with pooled sera from patients or healthy subjects that contains the primary antibodies against various targets (1∶200 dilution). Following washing, membranes were incubated for 1 hour at room temperature with horseradish peroxidase (HRP)-linked monoclonal anti-human immunoglobulin M (IgM) (1∶5000; Invitrogen, USA). The membranes were again washed and then visualized using chemiluminescence substrate (Pierce, USA) and 18 cm × 24 cm films (Kodak, USA).

### Functional annotation and protein interaction analyses

Functional annotation analysis was performed using DAVID v6.7 (Database for Annotation, Visualization, and Integrated Discovery), which provides a comprehensive set of functional annotation tools to understand the biological significance associated with large lists of genes or proteins [17]. This functional categorization is considered significant when a p-value of less than 0.05 (p<0.05) is obtained. STRING v9.1 (Search Tool for the Retrieval of Interacting Genes) was used to examine protein–protein interaction networks [18].

## Results and Discussion

### Image analysis of 2-DE serum protein profiles from healthy subjects and OSCC patients

**Figure 1:**
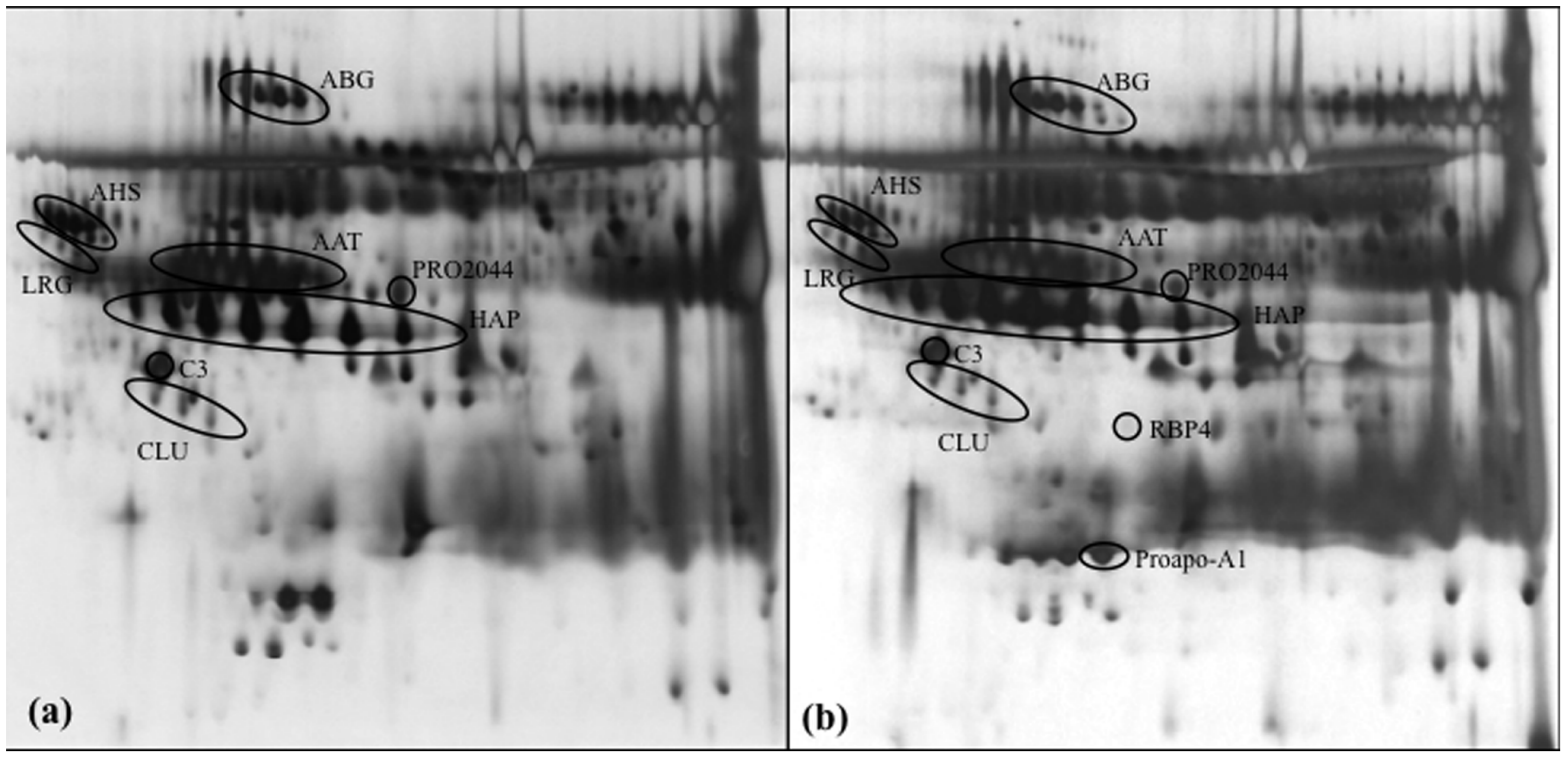
Representative 2-DE serum protein profiles of normal controls and OSCC patients. Unfractionated serum samples of (a) normal controls and (b) OSCC patients were subjected to 2-DE and silver staining. The labeled spot clusters are proteins which are consistently identified in profiles of normal controls and OSCC patients. α2-HS-glycoprotein (AHS) and α1-antitrysin (AAT) are high abundance proteins that typically appeared in protein profiles.

Unfractionated serum samples were separated via 2-DE to generate high-resolution proteome profiles for healthy subjects (Fig. 1a) and OSCC patients (Fig. 1b). Protein spots in the 2-DE gels from OSCC patients (n = 25) and normal controls (n = 25) were analyzed using PDQuest 2-D gel analysis software. This comparative analysis revealed several up- and down-regulated proteins in the serum of OSCC patients (Table 1).

**Table 1:** The relative expression of host specific proteins among the sera of patients.

Protein Entry Name	Protein Name	Fold Change
LRG	Leucine-rich alpha 2-glycoprotein precursor	0.21 (down)
ABG	Alpha-1-B-glycoprotein	0.45 (down)
CLU	Clusterin	0.60 (down)
PRO2044	PRO2044	0.63 (down)
HAP	Haptoglobin	1.47 (up)
Proapo-A1	Proapolipoprotein	1.82 (up)
RBP4	Retinol binding protein 4	2.66 (up)

Fold change measures the degree of change in the protein of the OSCC patients (n = 25) when compared to normal controls (n* = *25). This is measured by dividing the average spot intensity in the patients by the average spot intensity in the controls. (up) represents up-regulated expression whilst (down) represents down-regulated expression of protein spot.

### Identification of possible biomarkers using MS

Following digestion of the differentially expressed spots, MS analysis allowed us to identify the following seven host-specific proteins: leucine-rich α2-glycoprotein (LRG), alpha-1-B-glycoprotein (ABG), clusterin (CLU), PRO2044, haptoglobin (HAP), proapolipoprotein A1 (proapo-A1) and retinol-binding protein 4 precursor (RBP4) (Table 1). Furthermore, MS analysis revealed complement C3c (C3) as additional host-specific protein, which found to be immunoreactive and also five non-host factors (A1, A2, A3, A4, and A5), which corresponded to antigens from *Acinetobacter lwoffii, Burkholderia multivorans, Myxococcus xanthus, Laribacter hongkongensis*, and *Streptococcus salivarius*. In total, we identified 13 host and non-host specific protein spots, which were subsequently subjected to MS analysis (MALDI-TOF/TOF). Data regarding the identification (ID), MASCOT accession number, isoelectric point (pI), and molecular mass (Mr) for each protein are presented in Table 2 and 3.

**Table 2:** Mass spectrometric identification of host-specific protein spots from serum protein profiles using MASCOT search engine and the NCBI database.

Protein Name	MASCOT accessionnumber	pI	Theorecticalmass	Sequencecoverage	Searchscore	Queriesmatch	Expectedvalue
Alpha-1-B-glycoprotein – human [Homo sapiens]	gi|69990	5.65	52479	40%	824	24	9.1e-078
Leucine-rich alpha-2-glycoprotein precursor [Homo sapiens]	gi|1641846	6.45	38382	40%	601	18	1.8e-055
Clusterin [Homo sapiens]	gi|2666585	5.60	16267	11%	39	4	29
Retinol binding protein 4 [Homo sapiens]	gi|1808832	5.76	23371	48%	276	14	2.9e-021
PRO2044 [Homo sapiens]	gi|6650826	6.97	39984	45%	355	17	3.7e-029
Haptoglobin [Homo sapiens]	gi|3337390	6.14	38722	32%	264	11	4.6e-020
Proapollipoprotein [Homo sapiens]	gi|178775	5.45	28944	53%	503	20	1.1e-045
Chain B, Human Complement Component C3 [Homo sapines]	gi|7810126	5.55	114238	20%	469	24	1.5e-040

**Table 3:** Mass spectrometric identification of non-host specific protein spots from serum protein profiles using MASCOT search engine and the NCBI database.

Protein Name	MASCOT accessionnumber	pI	Theorecticalmass	Sequencecoverage	Searchscore	Queriesmatch	Expectedvalue
(A1) Predicted protein [*Acinetobacter lwoffii* SH145]	gi|262375905	8.89	9185	83%	56	12	30
(A2) Hypothetical protein BURMUCGD2M_4365 [*Burkholderia multivorans* CGD2M]	gi|221195969	8.31	4546	92%	51	7	1e+002
(A3) Hypothetical protein MXAN_1050 [*Myxococcus xanthus*DK 1622]	gi|108761930	5.05	10984	36%	52	8	82
(A4) Hypothetical protien LHK_003399 [*Laribacter* *hongkongensis* HLHK9]	gi|226939330	9.50	6340	68%	44	6	4.2e+002
(A5) Hemolysin A [*Streptococcus salivarius* SK126]	gi|228476878	5.47	30333	20%	50	10	1.2e+002

### Down-regulated host-specific proteins in OSCC patients

Comparing our candidate OSCC biomarkers to control samples, we found that LRG, ABG, CLU, and PRO2044 displayed 0.21-, 0.45-, 0.6-, and 0.63-fold down regulation, respectively (p<0.05). This indicated that CLU and PRO2044 represented the most significantly decreased protein spots. Notably, all of these down-regulated factors have been previously studied and could be important with regard to OSCC.

LRG is a protein that has been observed in patients with bacterial infections [19], severe acute respiratory syndrome [20], as well as various malignancies, including pancreatic [21], liver [22], and lung [23] cancers. Moreover, LRG has been suggested to play an anti-apoptotic role during stress. Nevertheless, Weivoda et al. [24] has reported low levels of LRG in patients with inflammatory arthritis, in spite of the fact that LRG can be produced in response to inflammation. Overall, the relevance of LRG in distinct patient groups remains ill defined.

ABG is one of the eight host-specific proteins observed in this study. Interestingly, decreased ABG expression has also been observed in pancreatic cancer [25]. On the other hand, up-regulated ABG was previously reported in bladder cancer [26], non-small cell lung cancer [27], and squamous cell carcinoma of the uterine cervix [28].

With regard to CLU, differential expression has been linked to oncogenesis and tumor growth in bladder [29], breast [30], colorectal [31], ovarian [14], pancreatic [32], and prostate [33] cancers. However, although many researchers have reported increased CLU expression during tumorigenesis, we have observed significantly down-regulated CLU levels in sera from OSCC patients. Nevertheless, other reports have also described a similar loss of CLU expression in various tumors, including prostate cancer [34], pancreatic cancer [32], esophageal squamous cell carcinoma [35] and neuroblastoma [36]. Notably, these discrepancies may stem from the fact that there are differentially expressed CLU isoforms in human tissues and fluids that may exhibit distinct functions in tumors [14], [37]. Even though it has been suggested that CLU down regulation could be associated with disease progression [32], [38], this may depend on the type of cancer [39], [40]. Notably, the true function of CLU has remained elusive despite extensive investigation. So far, CLU has been proposed to participate in the immediate cellular response to stress, which regulates cell growth and survival [41]. In this regard, its function appears isoform dependent, with both proapoptotic and anti-apoptotic forms [34].

Finally, our analysis revealed that PRO2044 (the C-terminal fragment of albumin, ALB) was the most down-regulated protein in the sera of OSCC patients. Interestingly, Kawakami et al. [22] observed that PRO2044 was also down regulated in hepatocellular carcinoma patients following curative radiofrequency ablation. In contrast, Jin et al. [42] reported that PRO2044 levels were increased in the cerebrospinal fluid of patients with Guillain–Barré syndrome, an acute inflammatory autoimmune disorder of the peripheral nervous system.

### Up-regulated host-specific proteins in OSCC patients

In addition to down-regulated proteins, up-regulated host-specific antigens were also identified in OSCC patients, including HAP, proapo-A1, and RBP4. Compared to the control samples, these proteins displayed 1.47-, 1.82-, and 2.66-fold increases, respectively (p<0.05). RBP4 was found to be the most up-regulated.

Notably, a correlation between HAP expression and malignancies has been reported [43], [44]. Indeed, similar to a previous study by Lai et al. [45], we found that HAP levels were significantly increased in sera from OSCC patients. Furthermore, since HAP is primarily hepatocyte-produced, a rise in its levels may indicate the occurrence of an acute phase response in OSCC [44]. Moreover, HAP has also been reported to participate in cell migration, extracellular matrix degradation, and arterial restructuring, suggesting its possible role in cancer [46]. In addition, HAP might act as an angiogenic agent that contributes to endothelial cell growth and differentiation [47].

With regard to proapo-A1 (or apo-A1), our findings are consistent with studies that have found elevated expression in various malignancies, including breast [48], colorectal [49], non-small cell lung [50] pancreatic [51], and hepatocellular [52] cancers. It is possible that increases in proapo-A1 might stem from reduced activity of proapo-A1 cleaving enzyme or higher turnover of apo-A1 [50], [53].

We also observed an elevation of RBP4 levels in OSCC. It has been suggested that RBP4 over expression in cancer cells could result from an inhibition of phosphatidylinositol-3 kinase (PI3K) activity [54], [55]. Moreover, RBP4 expression might be related to retinoid depletion, which is a common feature in cancer patients [56]–[58]. In addition, RBP4 levels can be influenced by transthyretin, which could reduce renal clearance of RBP4 [59].

### Non-host specific proteins detected in OSCC patients

In addition to the above down- and up-regulated self-antigens, we also identified five non-host markers in the proteomic profiles of OSCC patients. These proteins were derived from various bacteria, including *A. lwoffii, B. multivorans, M. xanthus, L. hongkogenisis*, and *S. salivarius*. Although our detection of these factors could have resulted from nosocomial infections [60], [61], heightened risk of malignancy has been linked to viruses, bacteria, and schistosomes [62], [63]. In fact, the relationship between bacterial infections and cancer has been discussed for decades [64], [65]. In this regard, there are several possible mechanisms by which bacteria could be oncogenic. For example, altered host responses during bacterial infection (e.g., chronic inflammation, antigen-driven lymphoproliferation, and hormone induction that promotes epithelial cell proliferation) have been suggested to influence oncogenesis [66]. Also, bacterial infection can lead to the production of toxin and/or carcinogenic metabolites that enhance oncogenesis [67]. In contrast, bacterial infections have also been suggested to play a protective role by altering host physiology and reducing cancer risk [68].

Oral cancer is considered to be a multi-factorial disease, as it can stem from exposure to several types of carcinogens, including microbial factors [69]. Indeed, several pieces of evidence have supported the association of microbial infection with oncogenesis. For instance, *Helicobacter pylori* has been linked to gastric cancer [70] and categorized by the World Health Organization International Agency for Research on Cancer (IARC) as a carcinogenic factor in humans [71]–[73]. Similarly, *Chlamydophila pneumoniae* has been associated with malignant lymphoma and lung cancer in males [74], [75], whereas *Candida albicans* and *Streptococcus anginosus* have been linked to oral carcinoma [70]–[76]. It has also been demonstrated that OSCC patients possess significantly elevated concentrations of certain bacteria in their saliva. Thus, changes in salivary microflora could represent a non-invasive diagnostic tool for predicting oral cancer [70].

### Confirmation of host-specific proteins by western blotting

Immunoglobulin M (IgM) antibodies are present in the circulation of normal humans and other mammalian species. IgM is initially secreted by B cells upon primary antigen stimulation [77], [78] and participates in natural defenses against foreign pathogens as well as neoplastic cells and tumors [79]. In fact, autoantibodies against specific cancer antigens have been identified for several types of tumors, including colon, breast, lung, ovary, prostate, and head and neck. These antibodies have been found to recognize several overexpressed (e.g., Her2), mutated (e.g., p53), or tissue-restricted (e.g., testis-cancer antigens) proteins, which are produced by cancer cells and elicit immune responses [6]. Therefore, detection of such autoantibodies in patient sera could be exploited as a means of cancer diagnosis. Indeed, the specificity and sensitivity of the antibody response to low antigen levels make it an ideal screening/diagnostic tool for early identification of cancer biomarkers in serum-based assays.

In order to extend the results obtained from our immunoproteomics analyses, we performed 2-DE immunoblots using OSCC patient and control sera (Table 4). In this regard, we performed immunoblots based on the following four conditions (i.e., categories 1–4): (1) normal pooled sera probed with normal pooled sera; (2) normal pooled sera probed with OSCC pooled sera; (3) OSCC pooled sera probed with normal pooled sera; and (4) OSCC pooled sera probed with OSCC pooled sera.

**Table 4:** Host- and non-host specific proteins on the 2-DE immunoblots.

Antigenic Proteins	Category			
	(a)	(b)	(c)	(d)
**1) Host specific proteins:**				
CLU	–	–	**/**	–
HAP	–	**/**	–	**/**
C3	–	–	**/**	**/**
Proapo-A1	**/**	–	**/**	**/**
RBP4	–	–	**/**	**/**
**2) Non-host specific proteins:**				
Predicted protein [*Acinetobacter lwoffii* SH145]	–	–	**/**	–
Hypothetical protein BURMUCGD2M_4365 [*Burkholderia multivorans* CGD2M]	–	–	**/**	–
Hypothetical protein MXAN_1060 [*Myxococcus xanthus* DK 1622]	–	–	**/**	–
Hypothetical protein LHK_0039 [*Laribacter hongkongenesis* HLHK9]	–	–	**/**	–
Hemolysin A [*Streptococcus salivarius* SK126]	–	–	**/**	–

/ Proteins of the patients or normal pooled serum recognized by the primary antibody.

– Proteins of the patients or normal pooled serum not recognized by the primary antibody.

**Figure 2:**
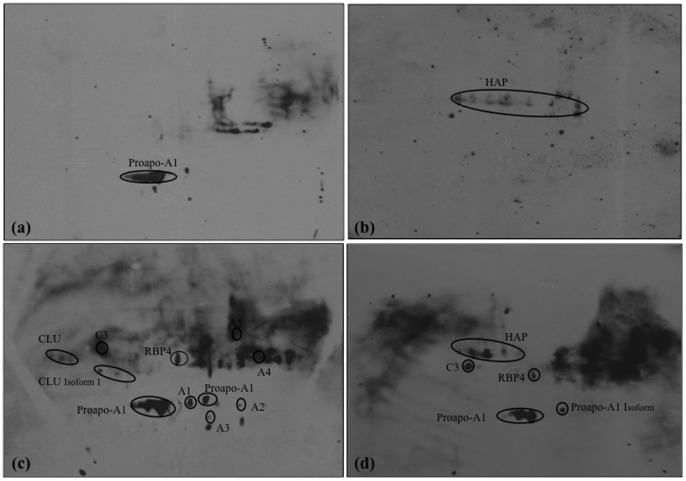
Results from 2-DE immunoblots for (a) normal pooled sera probed with normal pooled sera, (b) normal pooled sera probed with OSCC pooled sera, (c) OSCC pooled sera probed with normal pooled sera, (d) OSCC pooled sera probed with OSCC pooled sera. Unfractionated, pooled serum samples from control and OSCC patients were subjected to 2-DE and blotted onto nitrocellulose membranes, which were then probed with pooled sera and monoclonal anti-human IgM-HRP.

The use of normal pooled sera against normal and OSCC pooled sera was to prove that the reaction was restricted to the tumor specificity. Therefore, only few host-specific proteins could be detected in the normal controls. Based on our results, only proapo-A1 could be detected in category 1 (Fig. 2a), while HAP showed immunogenicity in category 2 blots (Fig. 2b). The detection of HAP in category 2 blot reveals the supportive role of the natural immunity against cancer cells apart from its involvement in the defense system against pathogens [79].

Based on the analysis in category 3 (Fig. 2C), our results show that the healthy control serums have the autoantibodies against OSCC serum antigens, CLU, C3, proapo-A1, and RBP4, which are the host-specific proteins that showed the most immunoreactivity in the immunoblot. These aberrant host-specific proteins were found to be more immunoreactive in the OSCC sera compared to the normal sera. In addition, other antigenic protein spots (A1, A2, A3, A4, and A5) were also detected in the category 3.

Finally, four reactive protein spots were observed in category 4, corresponding to C3, HAP, proapo-A1, and RBP4 (Fig. 2d). Therefore, the identified proteins are uniquely produced by the innate immune response of cancer cells. Altogether, five host-specific proteins were found to be immunoreactive in OSCC patients: CLU, C3, HAP, proapo-A1 and RBP4. Based on these analyses, our data support the rationale of using host-specific proteins as cancer biomarkers panel for early detection and diagnosis of OSCC [11].

### Functional annotation and protein interaction analysis

Functional annotation analysis was performed for our eight candidate host-specific biomarkers using DAVID v6.7 (http://david.abcc.ncifcrf.gov/) (Table 5). An analysis of biological processes revealed that HAP, apo-A1, and RBP4 are involved in homeostasis. Indeed, cancer-related inflammation is considered to be an essential hallmark of cancer due to the tumor-promoting consequences of inflammatory responses [80]. Thus, the study of aberrant homeostatic mechanisms has indicated that there exists an interaction between cancer cells and host immune cells during carcinogenesis.

**Table 5:** Functional annotation analysis of identified host-specific proteins using DAVID v6.7.

	Term Name	Enrichment Score+	Protein Count (%)	Protein Entry Name	p value
Biological Process	Chemical process	2.28	3 (37.5)	APOA1, HAP, RBP4	1.9e-2
	Homeostasis/Hemostasisprocess	2.28	3 (37.5)	APOA1, HAP, RBP4	4.0e-2
Cellular Component	Extracellular region	5.05	8 (100)	A1BG, ALB, APOA1, C3, CLU, HAP, LRG1, RBP4	2.4e-6
	Spherical high-density lipoproteinparticle	4.22	3 (37.5)	APOA1, CLU, HAP	7.2e-6
	High-density lipoprotein particle	4.22	3 (37.5)	APOA1, CLU, HAP	7.7e-5
	Plasma lipoprotein particle	4.22	3 (37.5)	APOA1, CLU, HAP	1.5e-4
	Protein-lipid complex	3.92	3 (37.5)	APOA1, CLU, HAP	1.5e-4

+ The classification stringency was set to high.

In addition, functional annotation analyses revealed that all of the host-specific proteins were located within the extracellular region and indicating that these proteins mediate immunogenic reactions against cancer cells. Therefore, this finding shows that these proteins could play an important role in the immunogenicity of carcinogenesis. Moreover, CLU, HAP, and apo-A1 were found to be associated with protein–lipid complexes that are responsible for cholesterol and lipid transportation. In this regard, cholesterol acts as a key contributor to carcinogenesis by promoting cell migration and mediating inflammatory processes. Indeed, lipoproteins may be the major suppliers of cholesterol to cancer cells via receptor-mediated mechanisms [81]. Therefore, alterations in these proteins could alter lipid metabolism, increasing the risk of cancer development and progression.

**Figure 3:**
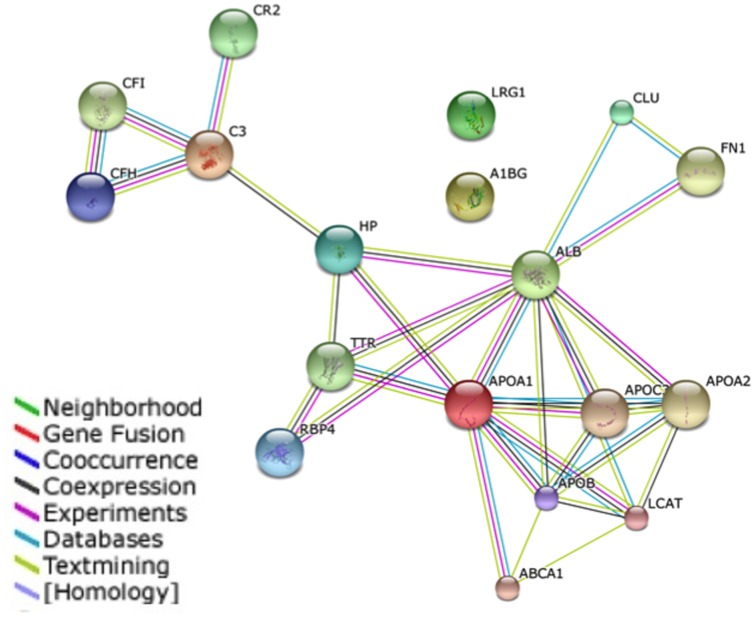
Interaction networks of identified host specific proteins using STRING v9.1. STRING database is a curated knowledge database of known and predicted protein-protein interactions. Most of the identified host-specific proteins have an established link with each other in the interaction network.

A protein interaction network was generated using the STRING v9.1 (http://string-db.org/) database to identify potential binding partners for our host-specific biomarkers (Fig. 3). Interestingly, most of the host-specific proteins could be linked to ten predicted functional partners (ABCA1, apo-A2, apo-B, apo-C3, CFH, CFI, CR2, FN1, LCAT, and TTR). Moreover, when KEGG pathway analysis was performed using DAVID v6.7, complement and coagulation cascades as well as the peroxisome proliferator-activated receptor (PPAR) signaling pathway could be linked to our identified host-specific proteins. Notably, these two pathways have been predicted to play important roles in cancer biology [82].

There were a total of four proteins (C3, CFH, CFI, and CR2) found to be involved in complement and coagulation cascades (p = 1.9×10^−4^). These pathways may participate in homeostatic processes that influence the host defense response. Indeed, the complement cascade is activated early in the immune response and might be important in cancer immunotherapy, as evidence has suggested that activation of complement regulators can promote tumor growth [83]. Activation of the coagulation cascade during cancer highlights how altered homeostasis can contribute to tumorigenesis. Indeed, it was reported that coagulation pathways could induce angiogenesis to facilitate tumor growth and metastasis [84]. As stated in the table 5 based on DAVID functional annotation analysis, “homeostasis” could be correlated with the proteins ApoA1, HAP and RBP4. However, it was reported that ApoA1, HAP have the possibility to relate with the process “hemostasis” [85], [86].

Furthermore, apo-A1, apo-A2, and apo-C3 were found to be associated with the PPAR signaling pathway (p = 6.1×10^−4^). PPARs are members of the nuclear hormone superfamily, which can be activated by fatty acids and their derivatives. The PPAR signaling pathway regulates lipid metabolism, cell proliferation, and cell differentiation. It has also been reported to be involved in the regulation of cancer cell apoptosis, proliferation, and differentiation. PPAR signaling is proposed to modify tumor growth by affecting angiogenesis, inflammation, and immune cell functions in the tumor cell environment [87]. Thus, targeting the PPAR signaling pathway could represent a potential strategy for cancer therapy.

## Conclusions

Using proteomic profiling, we were able to identify several differentially expressed host-specific proteins in the sera of OSCC patients, including LRG, ABG, CLU, PRO2044, HAP, proapo-A1, and RBP4. The immunogenicity of five of these proteins was further confirmed by western blot analyses (CLU, C3, HAP, proapo-A1 and RBP4). In addition, five non-host factors were detected, including proteins from *A. lwoffii, B. multivorans, M. xanthus, L. hongkogenisis*, and *S. salivarius*. As previously suggested [88], combined proteomic and serological approaches, such as the one used in the present study, can reflect numerous events occurring *in vivo* simultaneously due to the fact that patient serum is complex and consists of many proteins. Our data indicate that immunoproteomics approach could be a promising application for biomarker discovery and disease progression. Our study could be used as a landmark in a more comprehensive study and can be applied to individual patient serums or to a larger sample size. Using these methods, we have identified distinct serum biomarkers that might facilitate the development of early diagnostic tools for OSCC and promote further understanding the ‘host responses’ that occur in OSCC patients.
